# Supplementary figures and images for: Where talent flows: Trends and determinants of Chinese students’ city preferences
Source: PLoS One. 2026 Mar 5;21(3):e0343928. doi: 10.1371/journal.pone.0343928 (PMC12962534; doi:10.1371/journal.pone.0343928)

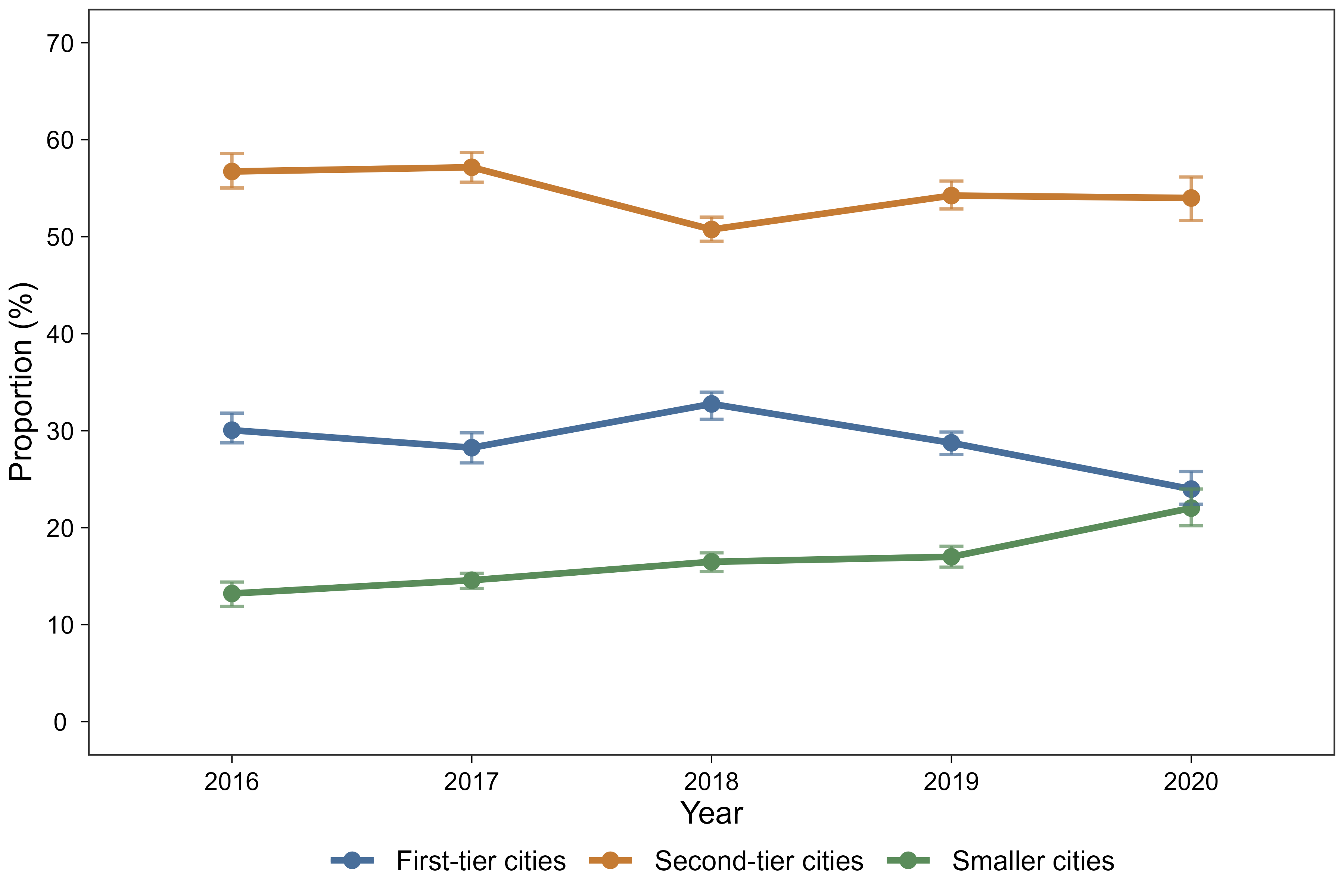

Supplement: S1 Fig — (TIF) [file pone.0343928.s001.tif]

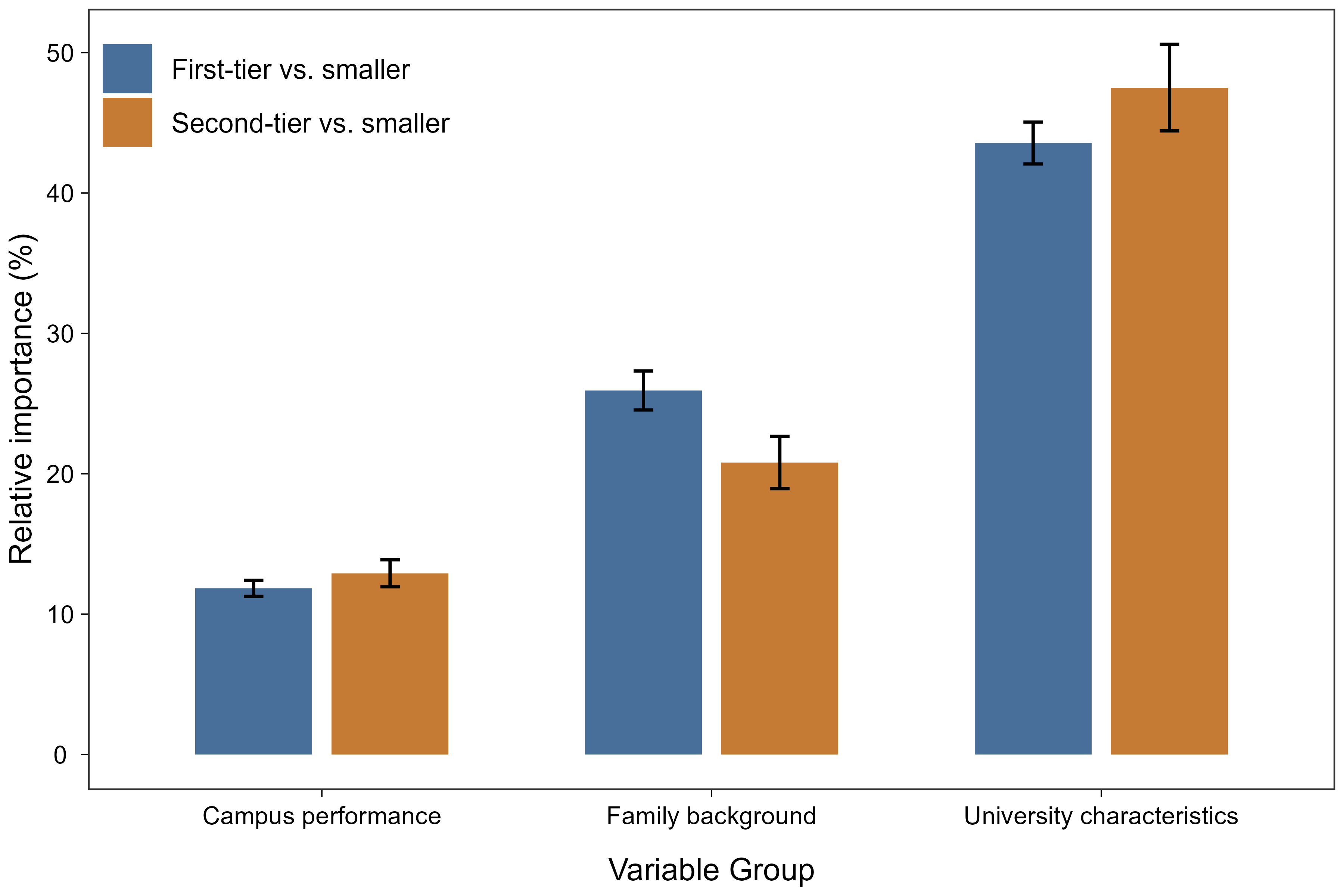

Supplement: S2 Fig — (TIF) [file pone.0343928.s002.tif]
