# Supplementary material for: Where talent flows: Trends and determinants of Chinese students’ city preferences
Source: PLoS One. 2026 Mar 5;21(3):e0343928. doi: 10.1371/journal.pone.0343928 (PMC12962534; doi:10.1371/journal.pone.0343928)
Supplement: S1 Table — (DOCX) [file pone.0343928.s003.docx]

**S1 Table. Comparison of variable distributions before and after missing-data deletion.**

| **Variable** | | **Category** | **Proportion difference (%)**  **(after - before)** | |
| --- | --- | --- | --- | --- |
|  |  |  |  |  |
| City preferences | | First-tier | -0.25 | |
|  |  | Second-tier | -0.05 | |
|  |  | Smaller | 0.30 | |
| **Campus performance** |  | | |  |
| Academic performance | | 1: Very poor | 0.42 | |
|  |  | 2: Poor | 0.30 | |
|  |  | 3: Average | 0.05 | |
|  |  | 4: Good | -0.59 | |
|  |  | 5: Excellent | -0.18 | |
| Leadership experience | | 0: No | 0.41 | |
|  |  | 1: Yes | -0.41 | |
| Extracurricular participation | | 0: No | 1.58 | |
|  |  | 1: Yes | -1.58 | |
| Party membership | | 0: No | 1.04 | |
|  |  | 1: Yes | -1.04 | |
| **Family background** | |  |  | |
| Urban *Hukou* | | 0: No | -1.29 | |
|  |  | 1: Yes | 1.29 | |
| Father’s education level | | 0: Primary | 0.23 | |
|  |  | 9: Junior high school | 0.60 | |
|  |  | 12: High school | -0.52 | |
|  |  | 15: Junior college | 0.36 | |
|  |  | 16: Bachelor | -0.63 | |
|  |  | 20: Master+ | -0.04 | |
| Father in public institutions | | 0: No | 0.36 | |
|  |  | 1: Yes | -0.36 | |
| Only-child status | | 0: No | 3.52 | |
|  |  | 1: Yes | -3.52 | |
| **University characteristics** | |  |  | |
| University type | | 1: Project “985” institutions | -1.05 | |
|  |  | 2: Project “211” institutions | -0.30 | |
|  |  | 3: Regular undergraduate colleges | -1.94 | |
|  |  | 4: Higher vocational institutions | 3.30 | |
| **Control variables** | |  |  | |
| Gender | | 0: Male | 0.71 | |
|  |  | 1: Female | -0.71 | |
| Degree level | |  |  | |
| Junior college | | 1 | 2.88 | |
| Bachelor | | 2 | -0.47 | |
| Master | | 3 | -2.37 | |
| Doctor | | 4 | -0.05 | |
| Geographic origin | |  |  | |
| West | | 0 | 1.02 | |
| East | | 1 | -0.91 | |
| Central | | 2 | 0.01 | |
| Northeast | |  | -0.11 | |
