# Supplementary material for: Where talent flows: Trends and determinants of Chinese students’ city preferences
Source: PLoS One. 2026 Mar 5;21(3):e0343928. doi: 10.1371/journal.pone.0343928 (PMC12962534; doi:10.1371/journal.pone.0343928)
Supplement: S2 Table — (DOCX) [file pone.0343928.s004.docx]

**S2 Table. Classification of cities into three tiers.**

| Classification | Cities |
| --- | --- |
| First-tier cities | Beijing, Shanghai, Guangzhou, Shenzhen |
| Second-tier cities | Shijiazhuang, Taiyuan, Shenyang, Changchun, Harbin, Nanjing, Hangzhou, Hefei, Fuzhou, Nanchang, Jinan, Zhengzhou, Wuhan, Changsha, Guangzhou, Haikou, Chengdu, Guiyang, Kunming, Xi'an, Lanzhou, Xining, Taipei, Hohhot, Nanning, Lhasa, Yinchuan, Ürümqi, Ningbo, Qingdao, Dalian, Xiamen, Suzhou, Wuxi, Changzhou, Foshan, Zhuhai, Zhongshan, Shaoxing, Jiaxing. |
| Smaller cities | Other cities |
