# Supplementary material for: Where talent flows: Trends and determinants of Chinese students’ city preferences
Source: PLoS One. 2026 Mar 5;21(3):e0343928. doi: 10.1371/journal.pone.0343928 (PMC12962534; doi:10.1371/journal.pone.0343928)
Supplement: S3 Table — (DOCX) [file pone.0343928.s005.docx]

**S3 Table. Variance inflation factors (VIF) for independent variables in the multinomial logistic regression model (full sample, 2016–2020).**

| **Variables** | **GVIF** | **Df** | **GVIF^(1/(2×Df))** |
| --- | --- | --- | --- |
| Academic performance | 1.11 | 4 | 1.01 |
| Leadership experience | 1.05 | 1 | 1.03 |
| Extracurricular participation | 1.06 | 1 | 1.03 |
| Party membership | 1.22 | 1 | 1.11 |
| Urban *Hukou* | 1.41 | 1 | 1.19 |
| Father’s education level | 1.36 | 5 | 1.03 |
| Father in public institutions | 1.18 | 1 | 1.09 |
| Log annual household income | 1.21 | 1 | 1.10 |
| Only-child status | 1.19 | 1 | 1.09 |
| University type | 2.29 | 3 | 1.15 |
| Year | 1.47 | 4 | 1.05 |
| Male | 1.04 | 1 | 1.02 |
| Degree level | 2.33 | 3 | 1.15 |
| Geographic origin | 1.29 | 3 | 1.04 |

**Notes**: GVIF is the generalized variance inflation factor. Df is the degrees of freedom for the variable (number of parameters estimated). GVIF^(1/(2×Df)) is adjusted GVIF for comparison across variables with different degrees of freedom.
