# Supplementary material for: Where talent flows: Trends and determinants of Chinese students’ city preferences
Source: PLoS One. 2026 Mar 5;21(3):e0343928. doi: 10.1371/journal.pone.0343928 (PMC12962534; doi:10.1371/journal.pone.0343928)
Supplement: S4 Table — (DOCX) [file pone.0343928.s006.docx]

**S4 Table. Year-specific adjusted GVIF values for independent variables (2016–2020).**

| **Variables** | GVIF^(1/(2×Df)) | | | | |
| --- | --- | --- | --- | --- | --- |
|  | 2016 | 2017 | 2018 | 2019 | 2020 |
| Academic performance | 1.02 | 1.01 | 1.01 | 1.01 | 1.01 |
| Leadership experience | 1.03 | 1.03 | 1.02 | 1.02 | 1.03 |
| Extracurricular participation | 1.02 | 1.04 | 1.04 | 1.03 | 1.02 |
| Party membership | 1.12 | 1.05 | 1.13 | 1.11 | 1.05 |
| Urban *Hukou* | 1.26 | 1.18 | 1.31 | 1.32 | 1.36 |
| Father’s education level | 1.04 | 1.03 | 1.05 | 1.04 | 1.05 |
| Father in public institutions | 1.20 | 1.01 | 1.21 | 1.21 | 1.23 |
| Log annual household income | 1.17 | 1.04 | 1.20 | 1.17 | 1.23 |
| Only-child status | 1.06 | 1.04 | 1.24 | 1.18 | 1.22 |
| University type | 1.22 | 1.11 | 1.20 | 2.25 | 1.07 |
| Male | 1.05 | 1.03 | 1.03 | 1.03 | 1.03 |
| Degree level | 1.19 | -- | 1.17 | 2.25 | -- |
| Geographic origin | 1.08 | 1.07 | 1.10 | 1.04 | 1.04 |

**Notes**: Degree level was excluded from the 2017 and 2020 models due to collinearity with university type. GVIF is the generalized variance inflation factor. Df is the degrees of freedom for the variable (number of parameters estimated). GVIF^(1/(2×Df)) is adjusted GVIF for comparison across variables with different degrees of freedom.
