# Supplementary material for: Where talent flows: Trends and determinants of Chinese students’ city preferences
Source: PLoS One. 2026 Mar 5;21(3):e0343928. doi: 10.1371/journal.pone.0343928 (PMC12962534; doi:10.1371/journal.pone.0343928)
Supplement: S5 Table — (DOCX) [file pone.0343928.s007.docx]

**S5 Table. Coefficient estimates of the multinomial logistic regression model for students’ employment city preferences (2016).**

| **Variables** | **First-tier vs. smaller** | **Second-tier vs. smaller** |
| --- | --- | --- |
| **Campus performance** |  |  |
| Academic performance (ref. = Very poor) |  |  |
| Poor | 0.26 (ns) | 0.33 (ns) |
| Average | 0.74** | 0.76** |
| Good | 1.04*** | 1.01*** |
| Excellent | 1.38*** | 1.08*** |
| Leadership experience (ref.= No) | 0.29** | 0.07 (ns) |
| Extracurricular participation (ref. = No) | 0.16 (ns) | 0.19 (*p* < 0.1) |
| Party membership (ref. = No) | -0.25 (*p* < 0.1) | -0.09 (ns) |
| **Family background** |  |  |
| Urban *Hukou* (ref. = No) | 0.10 (ns) | 0.15 (ns) |
| Father’s education level (ref. = Primary) |  |  |
| Junior high school | 0.17 (ns) | 0.38 (*p* < 0.1) |
| High school | 0.37 (*p* = 0.1) | 0.41* |
| Junior college | 0.21 (ns) | 0.38 (*p* < 0.1) |
| Bachelor | 0.94*** | 0.64* |
| Master+ | 1.54** | 1.04* |
| Father in public institutions (ref. = No) | -0.26* | -0.31** |
| Log annual household income | 0.15** | 0.10* |
| Only-child status (ref. = No) | -0.01 (ns) | 0.00 (ns) |
| **University characteristics** |  |  |
| University type (ref. = Project “985” institutions) |  |  |
| Project “211” institutions | 0.94*** | -0.52** |
| Regular undergraduate colleges | -1.79*** | -1.50*** |
| Higher vocational institutions | -0.13 (ns) | -0.14 (ns) |
| **Control variables** |  |  |
| Male (ref. = No) | 0.25** | 0.10 (ns) |
| Degree level (ref. = Junior college) |  |  |
| Bachelor | 1.02*** | 1.16*** |
| Master | 0.23 (ns) | 0.59*** |
| Doctor | 0.44 (ns) | 1.06* |
| Geographic origin (ref. = West) |  |  |
| East | 0.36** | 0.04 (ns) |
| Central | 0.68*** | 0.45*** |
| Northeast | 0.51* | 0.34 (ns) |

**Notes**: Different values represent standardized coefficients. Sample size: *N* = 9040. Model fit: *Log-Likelihood* = -7691.20, *McFadden R²* = 0.09, *Likelihood ratio test (χ²*) = 1511.10***. Significance levels: *** *p* < 0.001, ** *p* < 0.01, * *p* < 0.05.
