# Supplementary material for: Where talent flows: Trends and determinants of Chinese students’ city preferences
Source: PLoS One. 2026 Mar 5;21(3):e0343928. doi: 10.1371/journal.pone.0343928 (PMC12962534; doi:10.1371/journal.pone.0343928)
Supplement: S6 Table — (DOCX) [file pone.0343928.s008.docx]

**S6 Table. Coefficient estimates of the multinomial logistic regression model for students’ employment city preferences (2017).**

| **Variables** | **First-tier vs. smaller** | **Second-tier vs. smaller** |
| --- | --- | --- |
| **Campus performance** |  |  |
| Academic performance  (ref. = Very poor) |  |  |
| Poor | -0.09 (ns) | 0.17 (ns) |
| Average | 0.42 (ns) | 0.40 (ns) |
| Good | 0.76** | 0.64* |
| Excellent | 0.94** | 0.63* |
| Leadership experience (ref.= No) | 0.22** | 0.17* |
| Extracurricular participation (ref. = No) | 0.07 (ns) | 0.14 (ns) |
| Party membership (ref. = No) | -0.31* | -0.18 (ns) |
| **Family background** |  |  |
| Urban *Hukou* (ref. = No) | -0.01 (ns) | -0.12 (ns) |
| Father’s education level (ref. = Primary) |  |  |
| Junior high school | -0.51** | -0.14 (ns) |
| High school | -0.50* | -0.16 (ns) |
| Junior college | -0.52** | -0.29 (ns) |
| Bachelor | 0.14 (ns) | 0.24 (ns) |
| Master+ | 0.12 (ns) | -0.33 (ns) |
| Father in public institutions (ref. = No) | -0.07 (ns) | -0.06 (ns) |
| Log annual household income | 0.02 (ns) | 0.01 (ns) |
| Only-child status (ref. = No) | 0.09 (ns) | 0.18 (*p* < 0.1) |
| **University characteristics** |  |  |
| University type (ref. = Project “985” institutions) |  |  |
| Project “211” institutions | 1.22*** | -0.51** |
| Regular undergraduate colleges | -1.45*** | -1.25*** |
| Higher vocational institutions | -1.66*** | -1.57*** |
| **Control variables** |  |  |
| Male (ref. = No) | 0.06 (ns) | -0.08 (ns) |
| Geographic origin (ref. = West) |  |  |
| East | 0.77*** | 0.50*** |
| Central | 0.66*** | 0.54*** |
| Northeast | 0.96*** | 0.75*** |

**Notes**: Different values represent standardized coefficients. Sample size: *N* = 11888. Model fit: *Log-Likelihood* = -9767.80, *McFadden R²* = 0.11, *Likelihood ratio test (χ²*) = 2483.30***. Significance levels: *** *p* < 0.001, ** *p* < 0.01, * *p* < 0.05.
