# Supplementary material for: Where talent flows: Trends and determinants of Chinese students’ city preferences
Source: PLoS One. 2026 Mar 5;21(3):e0343928. doi: 10.1371/journal.pone.0343928 (PMC12962534; doi:10.1371/journal.pone.0343928)
Supplement: S7 Table — (DOCX) [file pone.0343928.s009.docx]

**S7 Table. Coefficient estimates of the multinomial logistic regression model for students’ employment city preferences (2018).**

| **Variables** | **First-tier vs. smaller** | **Second-tier vs. smaller** |
| --- | --- | --- |
| **Campus performance** |  |  |
| Academic performance  (ref. = Very poor) |  |  |
| Poor | 0.36 (*p* < 0.1) | 0.32 (*p* < 0.1) |
| Average | 0.43* | 0.43** |
| Good | 0.82*** | 0.67*** |
| Excellent | 0.92*** | 0.66*** |
| Leadership experience (ref.= No) | 0.38*** | 0.21** |
| Extracurricular participation (ref. = No) | 0.02 (ns) | 0.19* |
| Party membership (ref. = No) | -0.03 (ns) | 0.02 (ns) |
| **Family background** |  |  |
| Urban *Hukou* (ref. = No) | 0.15 (*p* < 0.1) | 0.06 (ns) |
| Father’s education level (ref. = Primary) |  |  |
| Junior high school | 0.14 (ns) | 0.67*** |
| High school | 0.33 (*p* < 0.1) | 0.78*** |
| Junior college | 0.32 (p<0.1) | 0.77*** |
| Bachelor | 0.76** | 1.02*** |
| Master+ | 1.01* | 0.68 (ns) |
| Father in public institutions (ref. = No) | -0.22* | -0.02 (ns) |
| Log annual household income | 0.23*** | 0.12** |
| Only-child status (ref. = No) | 0.11 (ns) | 0.09 (ns) |
| **University characteristics** |  |  |
| University type (ref. = Project “985” institutions) |  |  |
| Project “211” institutions | 1.23*** | 0.39* |
| Regular undergraduate colleges | -0.91*** | -0.87*** |
| Higher vocational institutions | -0.79*** | -1.09*** |
| **Control variables** |  |  |
| Male (ref. = No) | 0.34*** | 0.30*** |
| Degree level (ref. = Junior college) |  |  |
| Bachelor | 0.94*** | 0.50*** |
| Master | 0.20 (ns) | 0.23 (*p* < 0.1) |
| Doctor | 0.26 (ns) | 0.63 (ns) |
| Geographic origin (ref. = West) |  |  |
| East | 0.70*** | 0.06 (ns) |
| Central | 0.32** | -0.03 (ns) |
| Northeast | 1.00*** | 0.79*** |

**Notes**: Different values represent standardized coefficients. Sample size: *N* = 12477. Model fit: *Log-Likelihood* = -10909, *McFadden R²* = 0.09, *Likelihood ratio test (χ²*) = 2154.70***. Significance levels: *** *p* < 0.001, ** *p* < 0.01, * *p* < 0.05.
