# Supplementary material for: Where talent flows: Trends and determinants of Chinese students’ city preferences
Source: PLoS One. 2026 Mar 5;21(3):e0343928. doi: 10.1371/journal.pone.0343928 (PMC12962534; doi:10.1371/journal.pone.0343928)
Supplement: S8 Table — (DOCX) [file pone.0343928.s010.docx]

**S8 Table. Coefficient estimates of the multinomial logistic regression model for students’ employment city preferences (2019).**

| **Variables** | **First-tier vs. smaller** | **Second-tier vs. smaller** |
| --- | --- | --- |
| **Campus performance** |  |  |
| Academic performance  (ref. = Very poor) |  |  |
| Poor | 0.34 (*p* = 0.1) | 0.18 (ns) |
| Average | 0.74*** | 0.55*** |
| Good | 0.96*** | 0.67*** |
| Excellent | 1.28*** | 0.86*** |
| Leadership experience (ref.= No) | 0.38*** | 0.23** |
| Extracurricular participation (ref. = No) | 0.11 (ns) | 0.15 (*p* < 0.1) |
| Party membership (ref. = No) | -0.15 (ns) | -0.03 (ns) |
| **Family background** |  |  |
| Urban *Hukou* (ref. = No) | 0.27** | 0.18* |
| Father’s education level (ref. = Primary) |  |  |
| Junior high school | 0.38 (*p* < 0.1) | 0.56** |
| High school | 0.40 (*p* < 0.1) | 0.43* |
| Junior college | 0.56** | 0.59** |
| Bachelor | 1.18*** | 0.83*** |
| Master+ | 3.35** | 2.70** |
| Father in public institutions (ref. = No) | -0.19 (*p* < 0.1) | -0.06 (ns) |
| Log annual household income | 0.18*** | 0.12** |
| Only-child status (ref. = No) | 0.01 (ns) | 0.01 (ns) |
| **University characteristics** |  |  |
| University type (ref. = Project “985” institutions) |  |  |
| Project “211” institutions | 0.25 (*p* < 0.1) | -1.06*** |
| Regular undergraduate colleges | -1.87*** | -1.32*** |
| Higher vocational institutions | -1.22 (*p* = 0.1) | -1.55* |
| **Control variables** |  |  |
| Male (ref. = No) | 0.27*** | 0.19** |
| Degree level (ref. = Junior college) |  |  |
| Bachelor | 0.60 (ns) | 0.19 (ns) |
| Master | -0.15 (ns) | 0.04 (ns) |
| Doctor | -1.53 (*p* < 0.1) | -0.63 (ns) |
| Geographic origin (ref. = West) |  |  |
| East | 1.12*** | 0.42*** |
| Central | 1.06*** | 0.50*** |
| Northeast | 1.24*** | 0.90*** |

**Notes**: Different values represent standardized coefficients. Sample size: *N* = 11544. Model fit: *Log-Likelihood* = -9729.20, *McFadden R²* = 0.12, *Likelihood ratio test (χ²*) = 2557***. Significance levels: *** *p* < 0.001, ** *p* < 0.01, * *p* < 0.05.
