# Supplementary material for: Where talent flows: Trends and determinants of Chinese students’ city preferences
Source: PLoS One. 2026 Mar 5;21(3):e0343928. doi: 10.1371/journal.pone.0343928 (PMC12962534; doi:10.1371/journal.pone.0343928)
Supplement: S10 Table — (DOCX) [file pone.0343928.s012.docx]

**S10 Table. The complete set of coefficient estimates for all variables of the multinomial logistic regression model for employment city preference.**

| **Variables** | **First-tier vs. smaller** | **Second-tier vs. smaller** |
| --- | --- | --- |
| **Campus performance** |  |  |
| Academic performance (ref. = Very poor) |  |  |
| Poor | 0.17 (ns) | 0.19 (*p* < 0.1) |
| Average | 0.47*** | 0.44*** |
| Good | 0.78*** | 0.64*** |
| Excellent | 1.00*** | 0.70*** |
| Leadership experience (ref.= No) | 0.31*** | 0.18*** |
| Extracurricular participation (ref. = No) | 0.13** | 0.22*** |
| Party membership (ref. = No) | -0.10 (*p* = 0.10) | -0.00 (ns) |
| **Family background** |  |  |
| Urban *Hukou* (ref. = No) | 0.08* | 0.04 (ns) |
| Father’s education level (ref. = Primary) |  |  |
| Junior high school | 0.02 (ns) | 0.39*** |
| High school | 0.19* | 0.42*** |
| Junior college | 0.16 (*p* < 0.1) | 0.40*** |
| Bachelor | 0.88*** | 0.82*** |
| Master+ | 1.29*** | 0.81*** |
| Father in public institutions (ref. = No) | -0.04 (ns) | 0.03 (ns) |
| Log annual household income | 0.14*** | 0.08*** |
| Only-child status (ref. = No) | 0.09* | 0.12*** |
| **University characteristics** |  |  |
| University type (ref. = Project “985” institutions) |  |  |
| Project “211” institutions | 0.94*** | -0.39*** |
| Regular undergraduate colleges | -1.24*** | -1.07*** |
| Higher vocational institutions | -1.13*** | -1.16*** |
| **Control variables** |  |  |
| Year (ref. = 2016) |  |  |
| 2017 | 0.16** | 0.07 (ns) |
| 2018 | 0.01 (ns) | -0.16** |
| 2019 | -0.16** | -0.17*** |
| 2020 | -0.69*** | -0.45*** |
| Male (ref. = No) | 0.29*** | 0.18*** |
| Degree level (ref. = Junior college) |  |  |
| Bachelor | 0.23*** | 0.18*** |
| Master | -0.44*** | -0.06 (ns) |
| Doctor | -0.71* | 0.07 (ns) |
| Geographic origin (ref. = West) |  |  |
| East | 0.87*** | 0.34*** |
| Central | 0.68*** | 0.38*** |
| Northeast | 0.98*** | 0.71*** |

**Notes**: Different values represent standardized coefficients. Sample size: *N* = 50267. Model fit: *Log-Likelihood* = 43132, *McFadden R²* = 0.10, *Likelihood ratio test (χ²*) = 9169.80***. Significance levels: *** *p* < 0.001, ** *p* < 0.01, * *p* < 0.05.
