# Supplementary material for: Where talent flows: Trends and determinants of Chinese students’ city preferences
Source: PLoS One. 2026 Mar 5;21(3):e0343928. doi: 10.1371/journal.pone.0343928 (PMC12962534; doi:10.1371/journal.pone.0343928)
Supplement: Attachment — Submitted filename: Response to Reviewers.docx [file pone.0343928.s015.docx]

**REVIEWER COMMENTS TO THE AUTHOR**

**Reviewer #5:**

Summary

This manuscript uses five waves (2016–2020) of data from the Panel Study of Chinese University Students (PSCUS) to examine trends and determinants of Chinese graduates’ employment city preferences. By classifying cities into first-tier, second-tier, and smaller cities, and estimating multinomial logit models supplemented by a “bundled coefficient” approach, the authors compare the relative roles of campus performance, family background, and university characteristics. The study finds that university type and location are the strongest predictors of city preferences, while the influence of academic performance and leadership experience has become more pronounced over time, and graduates from non-elite institutions are more responsive to talent policies in second-tier cities. The topic is timely and relevant, the dataset is large and valuable, and the paper is generally clearly written. With some important clarifications and strengthening of the methods and interpretations, this study could make a useful contribution. Below are some major and minor comments that I hope will help improve the manuscript.

>> We sincerely thank you for this positive and encouraging evaluation of our study. We have carefully revised the manuscript to improve clarity, consistency, and transparency. The data structure and methods are now described in greater detail, terminology has been standardized throughout the text, and the limitations section has been expanded to clarify the scope of the analysis and the interpretation of results. Please see below for our detailed responses, point by point.

Major Comments

Comments 1: Nature of the data: panel vs. repeated cross-sections. The manuscript describes PSCUS as a “nationally representative longitudinal survey” and uses five waves of data, but it is not clear whether the analysis is based on true panel data (the same individuals followed over time) or on repeated cross-sections (different individuals in each wave). This distinction has direct implications for the modelling strategy, because if individuals appear in multiple waves, the independence assumption of the multinomial logit model is violated and standard errors may be underestimated.

Suggestions: Please clarify the exact structure of the analytic sample. In particular, you may wish to: (1) state explicitly whether individuals are re-interviewed across waves and, if so, how many unique individuals versus person-wave observations are included in the final N; (2) explain how you handle any within-individual correlation, for example by using panel or mixed-effects methods for multinomial outcomes, or by clustering standard errors at the individual level; (3) if instead the data are effectively repeated cross-sections, clarify this and briefly justify why panel methods are not required.

>> Thank you very much for this important comment. PSCUS is longitudinal in design, and some students may participate in multiple survey waves. However, the publicly available dataset used in this study is fully anonymized and does not include individual identifiers that allow respondents to be linked across waves. As a result, it is not possible to determine how many individuals are re-interviewed in multiple waves or to model within-individual correlation using panel or mixed-effects multinomial models. Given this data limitation, we treat the observations as pooled person-wave data and focus on population-level associations rather than individual-level trajectories, panel methods are therefore not applicable in this setting. To assess whether the pooled estimates are sensitive to temporal variation, we conducted additional multinomial logistic regressions separately for each survey wave. The direction and relative magnitude of key predictors remain largely stable across years (Tables S5-S9), suggesting that the main findings are not driven by any single wave.

We have clarified the structure of the analytic sample and the rationale for the modeling strategy in the revised *Data source* and *Statistical analysis* sections (Lines 101–115, 170–187).

Comment 2: Use of survey design information and national representativeness. The paper characterizes PSCUS as a nationally representative survey based on stratified sampling, yet the analysis does not indicate whether survey weights, stratification, or clustering are accounted for. If such design features exist but are not incorporated, this may weaken the claim of national representativeness and could affect the estimated associations.

Suggestions: It would be helpful to explain more clearly how the survey design is treated in the analysis. For example: (1) indicate whether PSCUS provides sampling weights and design variables, and whether these were used in the descriptive statistics and regression models; (2) if weights or design variables are available but not used, provide a brief rationale and consider softening statements about national representativeness; (3) if no weights are available, please state this explicitly and acknowledge it as a limitation.

>> Thank you very much for this helpful comment. PSCUS employs a stratified sampling design across university type, academic orientation, and geographic regions, and is designed to provide broad national coverage of Chinese university students. However, the publicly available PSCUS dataset does not provide sampling weights or detailed survey design variables (e.g., strata or primary sampling units), which limits the ability to conduct fully design-based, weighted analyses. Accordingly, survey weights are not incorporated in the analyses. To address concerns about sample composition, particularly the overrepresentation of Double First-Class universities, we conducted additional sensitivity analyses by resampling the data to match official national statistics on university types and refitting the models across multiple bootstrap iterations. The main patterns and conclusions remain robust. We have now explicitly acknowledged the lack of survey weights and design variables as a data limitation in the *Data limitation and policy implications* section (Lines 387–403) and clarified the treatment of survey design in the *Data source* section (Lines 101–115).

Comment 3: Dependent variable - preferences vs. actual choices. The key outcome variable is based on the survey question "Where would you most like to work after graduation?", which captures employment preferences or intentions rather than realized employment locations. However, in several places the manuscript uses terms such as "employment destinations", "city choices", or "job outcomes", which may give the impression that the analysis concerns actual post-graduation locations rather than intended ones.

Suggestions: To improve conceptual precision, it would be useful to: (1) consistently describe the outcome as employment city preferences or intended employment locations, and make clear early on that this reflects intentions rather than realised behaviour; (2) in the Discussion and Conclusions, soften causal language regarding "flows of talent" or "job outcomes" and frame the findings more explicitly as patterns of preference under conditions of uncertainty; (3) add a sentence or short paragraph in the limitations section noting that intentions may not fully translate into actual behaviour due to labour market constraints, family expectations, or other factors.

>> Thank you very much for this important clarification. Following this comment, we now consistently describe as “employment city preferences”, and we clarify early in the *Materials and methods* section that the survey question asks where students would most like to work after graduation (Lines 125–127). We have added a brief paragraph to the *Data limitation and policy implications* section noting that city preferences may not fully translate into actual behavior due to labor market constraints, institutional barriers, family considerations, or other unobserved factors. At the same time, we emphasize that employment preferences represent a meaningful and theoretically important precursor to actual mobility, as they reflect how individuals evaluate opportunities and constraints across cities prior to labor market entry (Lines 404–410).

Comment 4: Ethics statement and use of human data. In the submission form, the ethics statement is recorded as "N/A", and IRB approval and informed consent are listed as "Not applicable". At the same time, the study analyses individual-level survey data on university students, albeit in anonymized form and as secondary data from PSCUS. PLOS ONE typically expects a clear ethics statement for studies involving human data, even when formal approval is not required or the data are fully anonymized.

Suggestions: I would encourage you to provide a more explicit ethics description. For example: (1) clarify whether the original PSCUS data collection received ethical approval from an institutional review board or ethics committee, and how informed consent was obtained from participants; (2) if your own institution determined that secondary analysis of anonymized PSCUS data is exempt from additional IRB review, state this explicitly using wording consistent with PLOS ONE guidance, such as "The analysis was based on fully anonymized secondary data; according to the guidelines of [institution], additional ethical approval was not required."; (3) update the Ethics Statement and Methods sections so that the treatment of ethics and consent is transparent to readers.

>> Thank you very much for this important suggestion. The study is based on secondary analysis of the PSCUS dataset, which is publicly released for academic research use in fully anonymized form. We did not have access to any identifiable personal information, nor did we have any direct contact with survey participants. While detailed information on the original ethical review process of the PSCUS data collection is not publicly available, the survey was conducted by the Institute of Sociology at the Chinese Academy of Social Sciences as a large-scale institutional research project. According to the ethical guidelines, secondary analysis of fully anonymized data does not constitute human subjects research requiring additional IRB review. We have revised the *Institutional Review Board Statement* (Lines 488–492).

Comment 5: Data availability and access to PSCUS. There is a small inconsistency between the submission form and the manuscript regarding data availability. The submission form indicates that all data are within the manuscript and its Supporting Information files, whereas the manuscript states that the data are available from http://www.pscus.cn/. Since PSCUS is an external dataset that typically requires registration or permission, readers may not be able to access the raw microdata directly.

Suggestions: It would strengthen transparency to harmonise and specify the data availability information. In particular, you may wish to: (1) ensure that the Data Availability Statement in the manuscript and the text entered in the submission system are consistent; (2) clarify what exactly is contained in the Supporting Information (for example, summary tables, derived indicators, or analysis code) and what must be obtained directly from PSCUS (for example, the raw microdata); (3) indicate whether access to PSCUS is open or subject to application or approval, and, if the raw data cannot be shared publicly, follow the PLOS ONE template for restricted third-party data by naming the data owner and explaining how qualified researchers can request access.

>> Thank you very much for this important point. We have revised the *Data availability statement* in the manuscript (Lines 496–499) to clarify that the study is based on third-party data from the Chinese University Student Survey (PSCUS). The raw data are not publicly available and cannot be redistributed by us, as data ownership and access are determined by the data provider.

Comment 6: Interpretation of multinomial logit coefficients. In the Results and Discussion, some effects are described using phrases such as "increasing the likelihood by X%". For instance, academic performance is said to increase the likelihood of choosing first-tier or second-tier cities by specific percentages. However, the coefficients reported in Table 3 are log-odds, and strictly speaking exp(beta) represents an odds ratio rather than a percentage change in probability. The term "likelihood" is therefore somewhat ambiguous.

Suggestions: To avoid possible misunderstanding, you might consider: (1) when discussing regression results, either reporting odds ratios (exp(beta)) and explicitly referring to changes in odds, for example "the odds of preferring first-tier cities are X times higher"; (2) alternatively, computing average marginal effects and then describing changes in predicted probabilities, such as "academic performance is associated with a Y percentage point higher probability of preferring first-tier cities"; (3) avoiding expressions that can be interpreted as simple percentage changes in probability unless they are directly based on predicted probabilities from the model.

>> Thank you for this helpful comment. We have revised the *Results* section to avoid ambiguous expressions. We now consistently report and interpret effects using odds ratios (exp(β)) (Lines 244–285).

Comment 7: Bundled coefficient analysis - method and justification. The attempt to group predictors into domains (campus performance, family background, university characteristics) and to compare their relative contribution is conceptually appealing, but the current description of the "bundled coefficients" approach is quite brief. It is not entirely clear how the group-level effect values reported in Table 4 are calculated, whether raw coefficients or transformed values are used, whether they are weighted, and how their standard errors are obtained. The link to Frangioni (2002) also remains somewhat indirect for readers who are unfamiliar with bundle methods in optimisation.

Suggestions: It would be helpful to elaborate on this method in the Statistical Analysis section. Possible steps include: (1) formally defining how each group’s effect value is constructed from the underlying regression coefficients, including whether absolute values, squared coefficients, or other transformations are used; (2) explaining why this metric is appropriate for comparing "relative importance" across domains and how the associated standard errors are derived; (3) considering references to methodological work that deals directly with the relative importance of groups of predictors, such as R-squared decomposition, dominance analysis, or Shapley value methods, and positioning your approach relative to these; (4) at a minimum, presenting the bundled coefficient results as an exploratory summary of group contributions rather than a precise measure of explanatory power, and acknowledging this in the discussion of limitations.

>> Thank you for this comment. In the revised manuscript, we have expanded the *Statistical analysis* section to explicitly describe how the grouped effect measures are constructed (Lines 188–193). Specifically, all predictors are first standardized, and the absolute standardized coefficients within each conceptual domain (campus performance, family background, and university characteristics) are then averaged to produce a summary indicator of relative effect magnitude. This measure is not weighted and does not involve variance-based decomposition.

Comment 8: Causal language around policy interventions. The manuscript links changes after 2018 to intensified "talent war" policies in second-tier cities, including housing subsidies and hukou reforms. This interpretation is plausible and certainly interesting, but in the current design there are no explicit policy variables at the city level in the models, and the evidence relies on descriptive trends over time rather than a causal identification strategy.

Suggestions: To keep the conclusions aligned with the analytical design, you might: (1) soften statements that imply a direct causal effect of specific talent policies and instead present them as possible explanations or interpretations consistent with the observed patterns, using phrases such as "may be related to" or "is consistent with the interpretation that"; (2) briefly note that identifying the causal impact of concrete policy measures would require a different research design, for example incorporating city-level policy timing in a difference-in-differences framework, and mention this as a promising direction for future research.

>> Thank you for this thoughtful comment. we have further softened and clarified the policy-related interpretation in the *Discussion* by explicitly framing these observations as descriptive and interpretive rather than causal (Lines 429–432). We now state more clearly that the temporal alignment between changes in students’ employment city preferences and the intensification of local talent policies after 2018 is presented as a possible interpretation that is consistent with the observed patterns, rather than as evidence of policy effects (Lines 345–348). In addition, we have added a brief note indicating that identifying the causal impact of concrete policy interventions would require a different research design, which we highlight as a promising direction for future research (Lines 432–435).

Minor Comments

Comments 9: Terminology consistency. The manuscript alternates between expressions such as "employment city choices", "preferences", "performances", and "flows". Using several different terms for the same concept can be slightly confusing, especially since "performances" is more commonly associated with academic outcomes than with location preferences.

Suggestions: I suggest standardising the terminology as much as possible. For example, you could: (1) select one primary term, such as "employment city preferences" or "intended employment locations", and use it consistently throughout the text; (2) state clearly in the early part of the paper that these preferences refer to intended post-graduation locations rather than realised job placements.

>> We have standardized terminology throughout the manuscript, consistently using “employment city preferences” to refer to students’ intended employment locations. We also clarify in the *Data limitation and policy implications* section that these preferences reflect intentions rather than actual employment outcomes (Lines 404–410).

Comment 10: Table and figure labelling. In Table 4, the phrase "employment city performances" appears, which appears to be a typographical slip and may cause confusion. More generally, some readers may benefit from slightly more detailed captions.

Suggestions: You may wish to: (1) correct "performances" to "preferences" in Table 4; (2) check that all tables and figures are referenced in the main text in numerical order; (3) ensure that each caption is self-contained, briefly indicating the meaning of key variables and units so that readers can understand the content without returning to the main text.

>> We have corrected the typographical error in the Table and carefully checked the entire manuscript.

Comment 11: Description of recoding city tiers. Section 2.2.1 notes that response options differed slightly across survey waves and that you recoded them into three tiers, but the details of this recoding are not fully documented. For replication and substantive interpretation, readers may wish to know more about how cities were classified.

Suggestions: It would improve transparency to add a brief explanation of the recoding. For instance: (1) provide, either in the main text or in an appendix, a list or description of which specific cities fall into the first-tier, second-tier, and smaller city categories; (2) explain how borderline or changing categories were handled when harmonising response options across waves.

>> Thank you for this suggestion. To improve transparency, we have added an appendix (S2 Table) listing the specific first- and second-tier cities included in our analysis. Smaller cities are defined as all remaining other cities.

Comment 12: Language and style. The English writing is generally clear and readable, but there are a few minor grammatical issues, occasional repetition, and some long sentences that could be streamlined.

Suggestions: After substantive revisions are complete, a careful language edit would be beneficial. In particular, you may wish to: (1) correct instances such as "employment city performances" to "employment city preferences"; (2) check for consistent singular-plural agreement in phrases like "university characteristics... were/was"; (3) shorten a few very long sentences in the Discussion to improve clarity and flow.

>> Thank you for this suggestion. We have carefully reviewed the manuscript for language, grammar, and style.

Comment 13: Limitations section. The paper already discusses some limitations, but a few important aspects could be made more explicit to give readers a fuller sense of the study’s scope.

Suggestions: You might consider adding short statements noting that: (1) academic performance is self-reported rather than based on administrative grade records; (2) the analysis is based on preferences rather than actual employment locations, which may diverge when students enter the labour market; (3) any constraints related to survey weights, complex sampling design, or missing data handling, as discussed in earlier comments, may affect generalisability and should be kept in mind when interpreting the findings.

Overall, I find this study interesting and potentially valuable. Addressing the points above—especially regarding the nature of the data, survey design, ethics, and the interpretation of the models—would substantially strengthen the manuscript.

>> Thank you very much for this important comment. We now explicitly note that the analyses are based on employment city preferences rather than actual post-graduation locations (Lines 404–410), and that the publicly available PSCUS dataset does not include sampling weights or detailed survey design information (Lines 387–391). We have emphasized that these limitations may affect the generalizability of the findings and that interpretations regarding policy effects remain suggestive rather than causal (Lines 429–432).
