# Supplementary material for: Where talent flows: Trends and determinants of Chinese students’ city preferences
Source: PLoS One. 2026 Mar 5;21(3):e0343928. doi: 10.1371/journal.pone.0343928 (PMC12962534; doi:10.1371/journal.pone.0343928)
Supplement: Attachment — Submitted filename: Response to Editor.docx [file pone.0343928.s016.docx]

**Additional Editor Comments**

The revisions address many of the reviewers’ technical concerns, particularly with respect to data structure and terminology standardization.

To further strengthen the manuscript, however, the theoretical framework should be more explicitly anchored in established migration literature. Clarifying how your analysis extends existing debates—and where it departs from them—will help demonstrate the paper’s contribution more clearly.

Drawing on the broader migration and human capital literature, the hypotheses could be framed through various lenses. First, a Spatial Sorting Hypothesis, grounded in human capital theory, posits that high-achieving students are disproportionately attracted to first-tier cities to maximize returns on skills, with institutional prestige functioning as a dominant labor-market signal. Second, a Compensatory Resource Hypothesis, derived from social capital theory, suggests that family background—through economic and relational resources—buffers the high risks and costs of migration to competitive urban centers, particularly for graduates without elite institutional credentials. Third, a Policy-Induced Preference Hypothesis, informed by choice architecture literature, proposes that local interventions such as hukou reforms and housing subsidies reshape perceived opportunity structures, making second-tier cities more attractive alternatives for non-elite graduates. While these merely are some of the possibilities, it is crucial to link this research to the bigger migration literature.

The manuscript’s contributions should be articulated more explicitly. First, unlike the predominantly cross-sectional literature, this study leverages five waves of longitudinal data to show how urban preferences evolve in response to macro-level shocks, including the COVID-19 pandemic and shifting labor demand—an important and novel contribution. Second, it provides systematic evidence that the “Double First-Class” university designation produces a stratified migration hierarchy in which institutional signals often outweigh individual performance in access to top-tier urban labor markets. Third, the findings demonstrate that “talent war” policies have uneven effects across graduate groups: non-elite students are significantly more responsive to local incentives, offering a more nuanced account of how policy interventions shape regional talent allocation.

More clearly foregrounding these theoretical linkages and substantive contributions would substantially increase the manuscript’s impact.

>> Thank you very much for your encouraging feedback and for highlighting how to further enhance the impact of our work. We have carefully revised the manuscript by more clearly anchoring our theoretical framework in established migration literature. In response, we have mainly revised the *Introduction* and *Discussion* sections to address these comments (Lines 62–76, 328–388):

1. Clarify theoretical framework and hypotheses: We now linking our study to these established migration theories using three complementary hypotheses. Each hypothesis is directly linked to our empirical analyses, explaining how university prestige, individual performance, and family background shape students’ employment city preferences. we have restructured the *Introduction* and *Discussion* to foreground three primary hypotheses:
(1) Spatial Sorting Hypothesis: We now frame the influence of institutional prestige as a dominant labor-market signal that dictates a stratified migration hierarchy. We further discuss individual performance as a secondary, conditional sorting mechanism within this hierarchy.
(2) Compensatory Resource Hypothesis: We emphasize the role of family background (social capital) as a buffer for graduates, especially those from non-elite institutions, against the costs and risks of competitive urban centers.
(3) Policy-Induced Preference Hypothesis: We incorporate the lens of “choice architecture” to explain how local interventions (*Hukou*, housing subsidies) reshape perceived opportunities for non-elite graduates.
(4) We leverage our five-wave longitudinal analysis to discuss how shifts in urban preferences (especially among non-elite students) align with the “choice architecture” created by local talent-attraction policies (e.g., *Hukou* reforms, housing subsidies).
2. Substantive Contributions: We now highlight the contribution of leveraging five waves of longitudinal data (2016–2020) to observe how students’ employment city preferences evolve under macro-level shocks, including COVID-19 and shifts in labor demand. Our findings suggest that the “Double First-Class” designation plays a stronger role than individual performance in shaping access to first-tier urban markets. We also observe that non-elite students appear more responsive to local incentives, whereas elite graduates tend to remain aligned with traditional urban hierarchies. These observations provide insights into the interplay of institutional prestige, individual merit, family resources, and policy interventions in early-career mobility.
3. . Terminology and Theoretical Linkage: We have standardized the terminology throughout the manuscript (e.g., using “spatial sorting”, “compensatory resource”, and “policy-induced preference”). We also revised the Discussion headings to reflect these theoretical linkages, ensuring the analysis linked directly to the broader migration literature.
